# Type I IL-1 Receptor (IL-1RI) as Potential New Therapeutic Target for Bronchial Asthma

**DOI:** 10.1155/2010/567351

**Published:** 2010-07-05

**Authors:** Jyh-Hong Lee, Li-Chieh Wang, Hsin-Hui Yu, Yu-Tsan Lin, Yao-Hsu Yang, Bor-Luen Chiang

**Affiliations:** ^1^Department of Pediatrics, National Taiwan University Hospital, National Taiwan University College of Medicine, Taipei 100, Taiwan; ^2^Graduate Institute of Clinical Medicine, National Taiwan University College of Medicine, Taipei 100, Taiwan

## Abstract

The IL-1R/TLR family has been receiving considerable attention as potential regulators of inflammation through their ability to act as either activators or suppressors of inflammation. Asthma is a chronic inflammatory disease characterized by airway hyperresponsiveness, allergic inflammation, elevated serum total, allergen-specific IgE levels, and increased Th2 cytokine production. The discovery that the IL-1RI–IL-1 and ST2–IL-33 pathways are crucial for allergic inflammation has raised interest in these receptors as potential targets for developing new therapeutic strategies for bronchial asthma. This paper discusses the current use of neutralizing mAb or soluble receptor constructs to deplete cytokines, the use of neutralizing mAb or recombinant receptor antagonists to block cytokine receptors, and gene therapy from experimental studies in asthma. Targeting IL-1RI–IL-1 as well as ST2–IL-33 pathways may promise a disease-modifying approach in the future.

## 1. Introduction

Asthma is characterized by allergic inflammation of the airways with local infiltration of eosinophils, mast cells, and activated T helper lymphocytes [1]. The initial immune response responsible for this is the generation of allergen-specific CD4+ T helper-2 cells (Th2) that produce Th2 cytokines (IL-4, IL-5, IL-9, and IL-13), but not T helper-1 (Th1) cytokines (IL-2, IL-12, and interferon-*γ* [IFN-*γ*]). Overwhelming evidence in literature supports the concept that allergic inflammation is driven by an imbalance between Th1 and Th2 cytokines, favoring the Th2 immune response [1]. IL-4, together with IL-13, is required for Th2-cell development and is intimately involved in the regulation of immunoglobulin E (IgE) production by sensitized allergen-specific B cells, which is a fundamental mechanism in the pathogenesis of allergic asthma [2]. IL-5 is the principal cytokine involved in eosinophil growth, maturation, differentiation, survival, and activation [3, 4]. Cross-linking of allergen-specific IgE on mast cells and the activation of T cells and eosinophils during subsequent encounters with antigens stimulates the release of various preformed and newlysynthesized products, including histamines, cytokines, and chemokines. Together, these lead to characteristic airway changes that contribute to airway obstruction, airway hyperresponsiveness (AHR), goblet cell metaplasia, mucus overproduction, mucosal edema, and airway remodeling [5].

Pulmonary allergic inflammation can be induced in small rodents, such as BALB/c mice, is widely used as an experimental model for human asthma, and is central to the preclinical development of drug therapies [6]. One of the most common murine models of allergen-induced airway inflammation involves mouse sensitization using a small dose of a protein allergen followed by allergen challenge of the airways to induce pulmonary inflammation [7]. This is usually done by injecting the protein intraperitoneally along with an aluminum hydroxide as adjuvant to enhance the protein's immunogenicity [8]. After the immune system has had a chance to mount a reaction against the antigenic protein (sensitization) over several days, the animal receives further antigen exposure either directly to the lungs in the form of an aerosol or via postnasal drip following nasal instillation (challenge). This characteristically leads to AHR, lung eosinophilia, mucus hypersecretion, and increased IgE levels, which are all features commonly associated with human allergic asthma [5].

The IL-1 family has been involved in inflammatory and immunologic responses [9]. Members contain activators and suppressors of inflammation [9]. Interleukin-1 receptors (IL-1Rs) and Toll-like receptors (TLRs) are members of a large superfamily of phylogenetically conserved proteins involved in innate immunity and inflammation [10]. The common characteristics of these two receptor families include their presence in the cytoplasmic region of a conserved sequence called Toll/IL-1R (TIR) domain [9, 11]. The IL-1R/TLR-driven immune response also has an essential role in the induction and/or regulation of allergic inflammation and disease exacerbations. 

ST2, one member of the IL*‑*1 receptor family, was firstly identified as an orphan receptor in 1989 [12]. In 2005, the discovery of interleukin-33 (IL-33) as an ST2 ligand provided new insights into ST2 signalling pathway [13]. ST2/IL-33 signalling is involved in T-cell mediated immune responses, particularly Th2 cells and the production of Th2-associated cytokines [14].

Allergic asthma is used as an example of a chronic inflammatory disease to show how IL-1R/TLR-related pathway offer possibilities of therapeutic intervention. Due to similar roles in the pathogenesis of allergic inflammation, focus was on the IL-1RI-IL-1 and ST2-IL-33 pathways to review their potential as therapeutic targets for treating asthma [15]. Special attention was given to the experimental approach in validating the possibility of targeting IL-1R/TLR to explore emerging treatments.

## 2. IL-1RI-IL-1

IL-1R was first described and cloned in the family [11, 16–21]. The IL-1R type 1 (IL-1RI) contains three extracellular immunoglobulin (Ig) domains [9, 11]. A second chain, the IL-1 receptor accessory protein (IL-1RAcP) [22] has been reported. It is essential for the signal transduction for IL-1 and IL-33 and is highly homologous to IL-1RI. IL-1RAcP forms a heterodimer with either the IL-1RI or the IL-33R*α* chain (ST2) [9]. IL-1RI and IL-1RAcP *form* the receptor complex for IL-1 (both IL-1a and IL-1b) and binds naturally occurring IL-1 receptor antagonists (IL-1Ra) [23]. The Drosophila protein Toll has a cytosolic domain homologous in sequence to IL-1RI [24], which is called the TIR domain. It is also found in the cytoplasmic domains of each TLR, sometimes shortened to the Toll-IL-1 receptor domain [25]. The TIR domains of IL-1RI and the coreceptor IL-1RAcP are necessary for signal transduction.

### 2.1. Signaling Pathway

Detailed structures for IL-1 bound to the IL-1RI/IL-1RAcP complex have been discovered, as well as structures for IL-1RA bound to IL-1RI/IL-1RAcP [26, 27]. In crystallization studies, IL-1RI undergoes conformational change when binding IL-1*β* and allows IL-1RAcP to form the heterodimer [9]. The formation of an IL-1 receptor heterodimer complex results in the approximation of adjacent TIR domains. This complex recruits intracellular adapter molecules, including MyD88 (myeloid differentiation factor 88), IRAK (IL-1R associated kinase), and TRAF6 (tumor necrosis factor [TNF] receptor-associated factor 6], to activate signal transduction pathways such as nuclear factor-*κ*B (NF-*κ*B), AP-1 (activator protein-1), JNK (c-Jun N-terminal kinase), and p38 MAPK (mitogen-associated protein kinase) [21, 28]. 

In an animal model of asthma, persistent NF-*κ*B activation in the bronchi is driven by granulocytes *via *IL-1*β* and TNF-*α*, which both induce I*κ*B-*β* degradation, perpetuating the immune response in asthmatic airways [29, 30]. IL-1Ra binds tightly to IL-1RI and blocks the activity of either IL-1*α* or IL-1*β*. One of the binding sites of IL-1Ra binds to IL-1RI with high affinity such that the second binding site cannot recruit the IL-1RAcP [9].

### 2.2. Studies in IL-1RI/IL-1 Pathway-Deficient Mice

The critical role of IL-1/IL-1R1 in the development of allergic Th2 responses in both mild and more severe asthma has been studied [31]. The role of IL-1 in pulmonary immune responses in models of allergic asthma has been investigated using IL-1R1-deficient [IL-1R1 (−/−)] mice. Pulmonary eosinophilic inflammation, goblet cell hyperplasia, priming of CD4+ T cells in bronchial lymph nodes and their recruitment to the lungs, and antibody responses, including IgG, IgE, and IgA, are strongly reduced in IL-1R1 (−/−) compared to control BALB/c mice. In contrast, in a model of more severe asthma, eosinophilic inflammation, antibody responses, and CD4+ T cell priming in lymph nodes are comparable between IL-1R1 (−/−) and wild-type mice. These results suggest an important role of IL-1/IL-1R1 in developing allergic Th2 responses, but may not be necessary for severe allergic Th2 responses. 

Another study demonstrates that IL-1 plays important roles in the development of AHR by validating IL-1*α*, IL-1*β*, and the natural inhibitor of both molecules [32]. It demonstrates that ovalbumin (OVA)-induced AHR, OVA-specific T cell proliferation, IL-4 and IL-5 production by T cells, and IgG1 and IgE production by B cells, in IL-1*α*/*β*-deficient [IL-1*α* (−/−)/*β* (−/−)] mice is significantly reduced from levels seen in wild-type mice, whereas responses seen in IL-1RA (−/−) mice are profoundly exacerbated or enhanced. These observations indicate that IL-1 plays important roles in the development of AHR and in establishing an important balance between proinflammatory cytokines and their inhibitors in allergic airway disease [33].

### 2.3. Experimental Application in Targeting the IL-1RI/IL-1 Pathway

Using the asthma animal model, the effects of targeting the IL-1RI/IL-1 pathway are summarized in Table 1. Reagents used include recombinant adenovirus expressing human IL-1ra (Ad-hIL-1ra), recombinant human interleukin-1 receptor antagonist (rhIL-1ra), and neutralizing antibodies to both IL-1*β* and IL-1*α*. Following airway sensitization with ovalbumin, there is suppression of AHR, inflammatory infiltration, and IL-5 production after antigen challenge in mice expressing the IL-1Ra adenovirus [34]. Using guinea pigs sensitized with different allergens, two studies using rhIL-1ra also demonstrate reduced airway symptoms induced by allergen challenge [35, 36]. Regarding the effect on inflammation, there is decreased expression of adhesion molecules only in one study [36]. In mice treated with neutralizing anti-IL-1*β* antibodies, AHR to inhaled antigen is partially reduced but with a concomitant decrease in the expression of other adhesion molecules, as well as the suppression of IL-4 [37].

## 3. ST2-IL-33

IL-33, another IL-1-like cytokine, drives the production of Th2-associated cytokines from *in vitro* polarized Th2 cells. *In vivo*, IL-33 induces the expression of IL-4, IL-5, and IL-13 and leads to severe pathologic changes in mucosal organs [13]. Mice injected with human IL-33 exhibit impressive pathologic changes in the arterial walls, lungs, and intestinal tissues [13]. Of particular relevance to the concept of IL-33-driven Th2 response is the prominent eosinophilic infiltration in lung tissue. Airway smooth muscle cells have IL-33 expression in both the protein and mRNA levels. IL-33 expression increases in bronchial biopsies in asthmatic subjects compared to controls, as well as subjects with severe asthma [38].

IL-33 mediates its biologic effects via ST2, an IL-1 receptor-related protein specifically expressed on mast cells and Th2 lymphocytes [15] that has been shown to function as an important effector molecule of Th2 responses in some experimental settings, including mouse asthma models. IL-33 administration induces AHR and goblet cell hyperplasia through the induction of IL-4, IL-5, and IL-13 entirely independent of the acquired immune system. Administration of IL-33 induces AHR and goblet cell hyperplasia in the lungs in the absence of an adaptive immune system [39]. It stimulates mast cells into producing IL-13 in an Fc*ε*RI-independent manner [40]. 

A prior study has shown ST2 to be highly expressed on Th2 cells and it appears to play a role in Th2 cell activation [41]. The ST2 gene is a member of the IL-1 receptor family, producing a secreted soluble form, soluble ST2 (sST2), and a transmembrane form, ST2L [42]. The structure of ST2L is similar to that of IL-1 receptor type I (IL-1RI), which consists of three extracellular immunoglobulin domains and an intracellular Toll/IL-1 receptor domain. Although the extracellular domain is common to sST2 and ST2L, sST2 lacks the trans-membrane and intracellular Toll-interleukin-1 receptor domains [15]. 

Beyond its role as a therapeutic target, sST2 has also emerged as a disease biomarker. Previous studies in human patients and animal models have shown that the level of sST2 in sera is elevated in asthma [43, 44]. Therefore, it is suggested that sST2 also plays a critical role in Th2 cell-mediated diseases. Administering a recombinant sST2-Fc fusion protein or a sST2 expression vector to asthmatic mice effectively attenuates inflammatory responses and decreases Th2 cytokine production [45]. These therapeutic experiments indicate that sST2 negatively regulates Th2 cell-mediated immunologic responses, as opposed to ST2L.

### 3.1. Signaling Pathway

The ST2 receptor is similar to the IL-1RI and IL-18R*α* in that it is composed of three extracellular Ig domains and an intracellular Toll domain. T1/ST2-dependent IL-33 responses resemble classical IL-1-like signaling, consistent with IL-33 receptor signaling via the recruitment of a coreceptor, IL-1RAcP [28]. IL-33 forms a heterodimer complex with ST2 and IL-1RAcP for signal transduction [46, 47]. Thus, IL-1RAcP represents a shared co-receptor within the IL-1 family that is essential for IL-33 signaling via T1/ST2, aside from the IL-1 signaling. Binding of IL-33 to ST2 receptor activates NF-*κ*B and MAPKs, induces Th2 cytokine expression, and leads to severe pathologic changes in mucosal organs. ST2 can sequester TLR adaptor molecules such as MyD88 [48], while TRAF6 is a critical signal transducer in the IL-33 signaling pathway [49]. 

### 3.2. Studies in ST2/IL-33 Pathway-Deficient Mice

ST2 (−/−) mice *develop* reduced allergic airway inflammation compared to wild-type (WT) mice. This is associated with reduced differentiation of IL-5^+^ T cells. However, IL-4 and IL-13 levels are similar in WT and ST2 (−/−) mice. There is a less pronounced increase in total cell, macrophage, and eosinophil accumulation in the BAL fluids of ST2 (−/−) mice compared to WT mice [50]. These indicate that IL-33/ST2 signaling is an important pathway in allergic airway inflammation. IL-33 may be involved in lung macrophage activation in clinical asthma and may play a significant role in the amplification of alternatively activated macrophage (AAM) polarization and chemokine production, which contribute to both innate and Ag-induced airway inflammation [51].

Using a primary pulmonary granuloma model induced with *Schistosoma mansoni *eggs, Townsend et al. have demonstrated that granuloma formation, characterized by eosinophil infiltration, is abrogated in T1/ST2-deficient mice [52]. Naive immune cell populations, cytokine levels, Th1 and Th2 cell development, and total Ig isotype production are normal in T1/ST2-deficient mice. In the absence of T1/ST2-expression, induction of primary synchronous pulmonary granuloma formation and Th2 cytokine (IL-4 and IL-5) production occurs in response to the formation of secondary pulmonary granuloma. Such data demonstrates clearly that T1/ST2 expression plays a role in the development of Th2-like cytokine responses.

### 3.3. Experimental Application in Targeting the ST2/IL-33 Pathway

Using the asthma animal model, the effects of targeting the ST2/IL-33 pathway is summarized in Table 2. Reagents used include neutralizing antibodies against IL-33, neutralizing antibodies against T1/ST2, recombinant sST2, and recombinant ST2-expressing plasmid. Treatment with anti-IL-33 significantly reduces serum IgE secretion, the number of eosinophils and lymphocytes, and concentrations of IL-4, IL-5, and IL-13 in bronchioalveolar lavage fluid compared to administering a control antibody, which indicate that blocking IL-33 may be a new therapeutic strategy for allergic asthma [53]. Blocking IL-33–T1/ST2 signaling using an antibody against T1/ST2 abrogates persistent AHR, suggesting that the IL-33–T1/ST2 pathway is necessary not only in the development of an allergic response but also for its maintenance. Anti-T1/ST2 antibody also significantly reduces the expression of IL-4. In contrast, IL-13 levels are unchanged [54]. 

In a murine model of asthma, pretreatment with sST2 reduces IL-4, IL-5, and IL-13 production from IL-33-stimulated splenocytes [42]. *This indicates* that sST2 acts as a negative regulator of Th2 cytokine production and allergic airway inflammation modulates the biological activity of IL-33 signaling. Soluble ST2 directly binds to IL-33 and suppresses the activation of NF-*κ*B in EL-4 cells stably expressing ST2L, suggesting that the complex of sST2 and IL-33 fails to bind to ST2L. Enhanced expression levels of sST2 *are* also achieved by intravenous gene transfer, resulting in a drastic reduction in the number of eosinophils and in IL-4 and IL-5 levels in BAL fluid [44].

## 4. Conclusions and Future Perspectives

**Figure 1:**
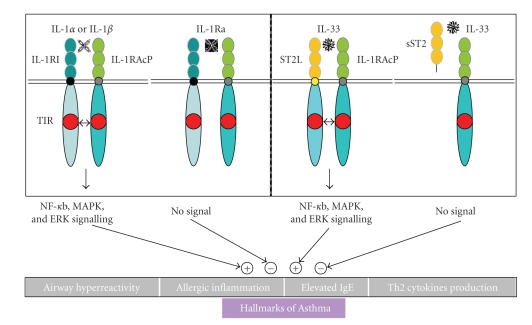
IL-1RI—IL-1 and T1/ST2—IL-33 pathways involved in allergic inflammation in asthma. “+” or “−” denote enhanced/activated or attenuated/suppressed effects of modulating signaling pathways, respectively. IL-1RI, IL-1 receptor type I; IL-1RAcP, IL-1 receptor accessory protein; IL-1Ra, IL-1 receptor antagonist; TIR, Toll/IL-1 receptor domains; sST2, soluble ST2; ST2L, trans-membrane ST2; NF-*κ*B, nuclear factor-*κ*B; MAPK, mitogen-activated protein kinase; ERK, extracellular signal*‑*regulated kinase. Modified from [13, 21, 29, 30].

Collectively, the IL-1RI−IL-1 and T1/ST2−IL-33 pathways are critical for immunologic control of allergic inflammation (Figure 1). From asthma animal studies, emerging strategies for asthma treatment are aimed at restoring the imbalance in cytokine (IL-1 and IL-33) and signaling pathways (IL-1RI and ST2) that mediate inflammatory and structural changes, and in skewing the cytokine profile away from a pro-inflammatory response towards a regulatory response. Potential therapeutic approaches may identify new strategies targeting key molecular mediators that drive inflammatory responses in asthmatic lungs. Such strategies include the depletion of cytokines using neutralizing mAb and soluble receptor constructs, receptor blocking via binding to recombinant receptor antagonists, small-molecule receptor antagonists or neutralizing mAb [55], and target receptors or cellular signal transduction pathways that are activated following cytokine receptor ligation, and gene therapy (expressing receptor antagonists or soluble receptor). Such approaches provide disease-modifying treatments.

Recently, there has been much focus on the signaling pathways involved in asthma. Among these, the MAPK pathway members JNK and p38 have attracted much interest, aside from NF-*κ*B, AP-1, and signal transducer and activator of transcription (STAT)-6 [56]. These signaling pathways are all involved in the IL-1RI—IL-1 and T1/ST2—IL-33 pathways. With advances in knowledge of cellular and molecular mediators involved in inflammation underlying asthma, there is much promise the future development of potential new therapeutics.

## Figures and Tables

**Table 1:** Effect of therapeutic experiments Targeting IL-1RI/IL-1 pathway.

Reagent	Mechanism	Animal model	airway hyperreactivity (AHR)	Inflammatory infiltration (eosinophils and lymphocytes)	IgE	Th1 cytokine	Th2 cytokine	Ref
Recombinant adenovirus expressing human IL-1ra (Ad-hIL-1ra)	receptor antagonist; gene therapy	Mice sensitized with ovalbumin (OVA)-immunized mice	↓	↓	N.A.	↑ (IFN-*γ*)	↓ (IL-5)	[34]

recombinant human interleukin-1 receptor antagonist (rhIL-1ra)	receptor antagonist	guinea pigs sensitized with Ascaris antigen	↓ (pulmonary resistance)	⟷	N.A.	N.A.	N.A.	[35]

recombinant human interleukin-1 receptor antagonist (rhIL-1ra);	receptor antagonist	asthmatic guinea pigs sensitized with ovalbumin (OVA)-immunized mice	the asthmatic symptom was obviously attenuated	↓ adhesionMolecules (sICAM-1 and P-selectin) levels	N.A.	N.A.	N.A.	[36]

antibodies against IL-1*β*	neutralizing monoclonal Ab	murine model of toluene diisocyanate-induced asthma	partial reduction	↓ adhesionMolecules (ICAM-1 and VCAM-1) levels	N.A.	N.A.	↓ (IL-4)	[37]

**Table 2:** Effect of therapeutic experiments Targeting ST-2/IL-33 pathway.

Reagent	Mechanism	Animal model	airway hyperreactivity (AHR)	Inflammatory infiltration (eosinophils and lymphocytes)	IgE	Th1 cytokine	Th2 cytokine	Ref
antibodies against IL-33	neutralizing monoclonal Ab	Mice sensitized with ovalbumin	N.A.	↓	↓	⟷	↓ (IL-4, IL-5, and IL-13)	[53]

antibodies against T1/ST2	blocking monoclonal	Mice sensitized with ovalbumin	↓	N.A.	N.A.	N.A.	↓ (IL-4); ⟷ (IL-13)	[54]

recombinant soluble ST2 protein	soluble recepto	Mice sensitized with ovalbumin; OVA-stimulated splenocytes	N.A.	N.A.	N.A.	↑ (IFN-*γ*) from stimulated splenocytes	↓ (IL-4, IL-5, and IL-13) from IL-33-stimulated splenocytes	[42]

plasmid expressing soluble ST2	soluble receptor; gene therapy	Mice sensitized with ovalbumin	N.A.	↓ (eosinophils)	N.A.	↑ (IFN-*γ*)	↓ (IL-4 and IL-5)	[44]
